# Poor infection prevention and control standards are associated with environmental contamination with carbapenemase-producing *Enterobacterales* and other multidrug-resistant bacteria in Swiss companion animal clinics

**DOI:** 10.1186/s13756-020-00742-5

**Published:** 2020-06-23

**Authors:** Janne S. Schmidt, Stefan P. Kuster, Aurélien Nigg, Valentina Dazio, Michael Brilhante, Helene Rohrbach, Odette J. Bernasconi, Thomas Büdel, Edgar I. Campos-Madueno, Stefanie Gobeli Brawand, Simone Schuller, Andrea Endimiani, Vincent Perreten, Barbara Willi

**Affiliations:** 1 Clinic for Small Animal Internal Medicine, Vetsuisse Faculty, University of Zurich, Zurich, Switzerland; 2 Division of Infectious Diseases and Hospital Epidemiology, University and University Hospital of Zurich, Zurich, Switzerland; 3 Institute of Veterinary Bacteriology, Vetsuisse Faculty, University of Bern, Bern, Switzerland; 4 Division of Small Animal Internal Medicine, Department of Clinical Veterinary Medicine, Vetsuisse Faculty, University of Bern, Bern, Switzerland; 5 Graduate School of Cellular and Biomedical Sciences, University of Bern, Bern, Switzerland; 6 Small Animal Clinic, Department of Clinical Veterinary Medicine, Vetsuisse Faculty, University of Bern, Bern, Switzerland; 7 Institute for Infectious Diseases, Faculty of Medicine, University of Bern, Bern, Switzerland

**Keywords:** Infection prevention and control, Small animal clinic, *Enterobacteriaceae*, Antimicrobial resistant pathogens, Multidrug-resistant pathogens, Colonization, Zoonosis

## Abstract

**Background:**

Intensive medical care in companion animal clinics could pose a risk for the selection and dissemination of multidrug-resistant organisms (MDROs). Infection prevention and control (IPC) concepts are key measures to reduce the spread of MDROs, but data on IPC standards in companion animal clinics is sparse. The study assessed IPC standards in seven companion animal clinics and practices in Switzerland by structured IPC audits and combined results with environmental MDRO contamination and MDRO carriage of the personnel.

**Methods:**

IPC audits were held between August 2018 and January 2019. The observations in 34 IPC areas were scored based on predefined criteria (not fulfilled/partially fulfilled/fulfilled = score 0/1/2). Environmental swabs and nasal and stool samples from veterinary personnel were tested for methicillin-resistant (MR) staphylococci and macrococci and for colistin-resistant, extended-spectrum β-lactamase- and carbapenemase-producing (CP) *Enterobacterales* (CPE). Species was identified by MALDI-TOF MS, antimicrobial resistance determined by microdilution and β-lactam resistance gene detection, and genetic relatedness assessed by REP−/ERIC-PCR and multilocus sequence typing.

**Results:**

Of a maximum total IPC score of 68, the institutions reached a median (range) score of 33 (19–55). MDROs were detected in median (range) 8.2% (0–33.3%) of the sampling sites. Clinics with low IPC standards showed extensive environmental contamination, i.e. of intensive care units, consultation rooms and utensils. CPE were detected in two clinics; one of them showed extensive contamination with CP *Klebsiella pneumoniae* (ST11, *bla*_OXA-48_) and MR *Staphylococcus pseudintermedius* (ST551, *mecA*). Despite low IPC scores, environmental contamination with MDROs was low in primary opinion practices. Three employees were colonized with *Escherichia coli* ST131 (*bla*_CTX-M-15_, *bla*_CTX-M-27_, *bla*_CTX-M-14_). Two employees carried CP *E. coli* closely related to environmental (ST410, *bla*_OXA-181_) and patient-derived isolates (ST167, *bla*_NDM-5_). MR *Staphylococcus aureus* (ST225, *mecA*) and MR *S. pseudintermedius* (ST551, *mecA*) of the same sequence types and with similar resistance profiles were found in employees and the environment in two clinics.

**Conclusions:**

The study indicates that IPC standards in companion animal clinics are variable and that insufficient IPC standards could contribute to the evolution of MDROs which can be transferred between the environment and working personnel. The implementation of IPC concepts in companion animal clinics should urgently be promoted.

## Background

Antimicrobial resistance (AMR) is an increasing challenge in human and veterinary health care and an emerging threat for public health [1]. Because multidrug-resistant (MDR) organisms (MDROs) are exchanged between humans, animals and the environment, the combat of AMR needs to be done under a One Health approach [2]. In addition to antimicrobial misuse and overuse in human and veterinary medicine, health care-associated transmission is regarded as a third main driver in the development and spread of AMR [3]. Infection prevention and control (IPC) concepts are well established in human health care settings to reduce the risk of transmission and spread of MDROs [4].

Progress in the area of small animal intensive care has led to the establishment of large specialized clinics in industrial countries in Europe, the United States and Asia. In these settings, the development and transmission of MDROs is facilitated by: 1) the high density of patients susceptible for infections, 2) daily invasive health care interventions, 3) the high percentage of patients receiving antimicrobial therapy, and 4) the use of last-generation and highest priority critically important antimicrobials [5, 6]. Accordingly, outbreaks with methicillin-resistant (MR) *Staphylococcus aureus* (MRSA) and *Staphylococcus pseudintermedius* (MRSP) or highly resistant *Acinetobacter* spp. isolates in small animal clinics in Europe have been documented [7–10]. Most recently, an outbreak involving CP *Escherichia coli* was reported in a large companion animal clinic in Switzerland [11]. Because of the close contact of dogs and cats with their owners [12], the acquisition of MDROs by companion animals could pose a considerable risk to humans [13–15]. Zoonotic transmission of MDROs from pets to humans have been proposed for MRSA, MRSP, and extended-spectrum β-lactamase (ESBL) and carbapenemase-producing (CP) *Enterobacterales* (CPE) [16–22]. Furthermore, MR coagulase-negative staphylococci (MRCoNS) with the same pulsed-field gel electrophoresis patterns were found in horses, personnel and environmental sites in an equine hospital in Denmark [23]. This finding suggests that MRCoNS strains can be shared between horses and personnel. MRCoNS represent an emerging cause of opportunistic infections in humans [24], and infections with MRCoNS have been documented in companion animals [25]. Recently, a novel *Macrococcus* spp., namely *Macrococcus canis*, has been isolated from healthy dogs and from infection sites of dogs; some isolates exhibited a MDR pattern [26, 27]. *M. canis* was found to have the ability to acquire AMR including methicillin resistance [28], but the potential contamination of the clinical environment with MR *Macrococcus* spp. has not yet been assessed.

Despite the key role of IPC concepts in limiting the dissemination of MDROs in human health care settings, data on IPC standards in companion animal veterinary institutions is sparse. The aim of the present prospective study was to evaluate IPC standards in different types of companion animal clinics and practices in Switzerland using structured IPC audits. Results were combined with investigations of the clinical environment and working staff for the presence or carriage of MR staphylococci, ESBL-producing, CP and colistin-resistant (COL-R) *Enterobacterales* and MR *Macrococcus* spp.

## Methods

### Aim, study design and IPC audits

The present prospective study was part of a large nation-wide project assessing the role of companion animal clinics in the dissemination of MDROs.

Three large clinics (A–C), two medium-sized clinics (D–E) and two first opinion practices (F–G) for companion animals were recruited. The institutions were chosen to be located in different parts of Switzerland and to cover different clinic/practice types (Additional file 1). One structured one-day audit per clinic or practice was performed between August 2018 and January 2019 by infectious disease specialists from human and small animal medicine to evaluate IPC standards. The audits covered 11 areas and 34 topics of IPC in small animal veterinary medicine [29, 30] and the observations were recorded and scored based on the criteria specified in Additional file 2. A score per IPC area and a total IPC score were calculated for each companion animal clinic and practice. Participation in the study was voluntary and was not reimbursed. The participating clinics and practices received a written report of the audits with suggestions for areas of improvements.

### Environmental sample collection

Samples from high-touch surfaces were collected in the institutions between June and November 2018. Sample collection was performed during a one-day visit from a list of high-touch surfaces (Additional file 3) including 69 sampling sites in large clinics (A–C) and 49 sampling sites in medium-sized clinics and practices (D–G). Differences in the number of resulting sampling sites at the different institutions were due to differences in infrastructure. The sampled surfaces were not disclosed prior to sampling. Samples were collected using transport swabs with enclosed tube containing Amies transport medium (Sarstedt AG & Co. KG, Nümbrecht, Germany). Environmental samples were tested for MRSA, MRSP, MRCoNS, MR *Macrococcus* spp., and ESBL-producing, CP and COL-R *Enterobacterales* (see below). In Clinic B, an additional 58 swabs were collected by the staff of the clinic in August 2018 from known high-risk areas (emergency room, *n* = 17; intensive care unit (ICU), *n* = 25; wards, *n* = 10; consultation room, *n* = 1; elevator, *n* = 1; lingerie, *n* = 2; entrance, *n* = 1; thermometers (10 pooled), *n* = 1) and analyzed specifically for CP *Enterobacterales*.

### Sample collection from employees

Veterinary employees in Clinics A–C and Practice G were recruited for the study and instructed by an information session held at the according institution. Participating staff self-collected a stool and a mid-turbinate nasal sample using a sampling kit (swabs and transport tubes containing Amies transport medium, Sarstedt AG & Co. KG; stool sample collection kit, Novoglas Labortechnik Langenbrinck, Niederrohrdorf, Switzerland). The samples were put into a transport container and sent to the Institute for Infectious Diseases, University of Bern, Switzerland for analysis. Nasal swabs were analyzed for MRSA, MRSP and MRCoNS, and the stool samples for COL-R *Enterobacterales*, and ESBL-producing and CP *Enterobacterales* as described below. The participants filled a questionnaire to obtain data on age, sex, clinic/practice type, working division and position, working duties, pet ownership, diet, medical history, medical and antibiotic treatment in the past, contact to the human health care system and to MDRO-carriers, and leisure and travel activities in order to evaluate risk factors for MDRO carriage. The questionnaire was pre-labelled with the pseudonymization number.

### Isolation and identification of the strains

MRSA, MRSP, MRCoNS and MR *Macrococcus* spp. were cultured using a two-step enrichment procedure and selected on chromogenic selective MRSA agar plates (BBL CHROMagar MRSA II, Becton Dickinson GmbH, Heidelberg, Germany) [31].

COL-R, 3rd generation cephalosporin-resistant and carbapenem-resistant *Enterobacterales* including *E. coli* and *Klebsiella* spp. were isolated after overnight enrichment in Luria-Bertani broth on specific selective plates including ChromID ESBL, ChromID CARBA SMART (bioMérieux, Suisse S.A., Geneva, Switzerland), and CHROMAgar plates supplemented with Colistin (bioMérieux). Colonies were purified onto MacConkey II Agar (Becton Dickinson GmbH) and identified to the species level by matrix-assisted laser desorption/ionization time-of-flight mass spectrometry (MALDI-TOF MS) analysis (Microflex LT, Bruker Daltonics GmbH, Bremen, Germany).

### Antibiotic susceptibility testing and strain typing

Antimicrobial susceptibility was determined by the measurement of the minimal inhibitory concentrations (MICs) of different antibiotics by broth microdilution using Sensititre EUST, EUVSEC, EUVSEC2 and GNX2F plates (Thermo Fisher Scientific, Waltham, USA). For *Staphylococcus* and *Macrococcus* species, MICs of 18 antimicrobials as specified in Table 1 were determined. MIC results were interpreted using the European Committee on Antimicrobial Susceptibility Testing (EUCAST) criteria [32], except for kanamycin and sulfamethoxazole, for which the Clinical and Laboratory Standards Institute (CLSI) criteria were used [33]; no clinical breakpoints were available for streptomycin. For the *Enterobacterales*, MICs of 14 antimicrobials as specified in Table 2 were determined. MIC results were interpreted using the EUCAST criteria [32], except for nalidixic acid, sulfamethoxazole and tetracycline, for which criteria from the CLSI were used [33]; no clinical breakpoints were available for azithromycin. The methicillin resistance genes *mecA*, *mecB*, and *mecD* were identified by PCR as previously described [34–36]. The ESBL and carbapenemase genes were identified using the CT103XL microarray (Check-Points, Wageningen, The Netherlands). Genetic relationships and clonality between isolates of the same species were determined by REP−/ERIC-PCR [37, 38] and by multilocus sequence typing (MLST) for MRSA, MRSP, MRCoNS, MR *Macrococcus* spp. using the corresponding schemes published in the PubMLST database (https://pubmlst.org/databases/) and for COL-R, ESBL-producing and CP *Enterobacterales* using the Center for Genomic Epidemiology (http://www.genomicepidemiology.org/).

**Table 1:** Characteristics and antibiotic resistance profile of the Gram–positive methicillin- and multidrug-resistant organisms

Insti-tution	Species^a^	Representative strain	Origin (n=)		ST^b^	Mec	Antibiotic resistance profile^c^																	
							CLI (>0.5)	TET (>2)	RIF (>0.5)	STR NA^d^	FUS (>1)	PEN (>0.125)	CHL (>8)	KAN (≥64)	SYN (>2)	VAN (>2/>4)^e^	GEN (>1)	TMP (>4)	ERY (>2)	CIP (>1)	FOX (screen ≥8)	LZD (>4)	MUP (≥256)	SMX (≥512)
			Env	Empl																				
**A**	*S. aureus*	18/EPI2716		1	398	A	≤0.12	**>16**	≤0.016	16	≤0.5	**>2**	8	**>64**	≤0.5	≤1	**8**	**>32**	≤0.25	≤0.25	**16**	2	≤0.5	≤64
	*S. aureus*	19/EPI0128		1	398	A	≤0.12	≤0.5	≤0.016	8	≤0.5	**0.5**	8	≤4	≤0.5	≤1	≤1	≤2	**>8**	≤0.25	4	2	≤0.5	≤64
	*S. aureus*	19/EPI0127		1	7	A	≤0.12	≤0.5	≤0.016	8	≤0.5	**>2**	8	≤4	≤0.5	≤1	≤1	≤2	≤0.25	≤0.25	4	2	≤0.5	≤64
	*S. aureus*	19/EPI0156		1	45	A	≤0.12	≤0.5	≤0.016	8	≤0.5	≤0.12	8	≤4	≤0.5	≤1	≤1	≤2	≤0.25	≤0.25	4	2	≤0.5	≤64
	*S. pseud-intermedius*	18/EPI2651	1		1338	A	**>4**	**>16**	≤0.016	**>32**	≤0.5	**>2**	**64**	**>64**	1	≤1	**4**	**>32**	**>8**	**>8**	**8**	≤1	≤0.5	**512**
	*S. haemolyticus*	19/EPI0140	1		30	A	0.5	≤0.5	≤0.016	≤4	≤0.5	**1**	≤4	≤4	≤0.5	≤1	≤1	≤2	≤0.25	**8**	**8**	≤1	≤0.5	128
	*S. haemolyticus*	19/EPI0129	1		68	A	**2**	**8**	>0.5	**>32**	**4**	**>2**	≤4	32	**4**	≤1	**8**	≤2	1	**2**	**>16**	≤1	≤0.5	**>512**
	*S. epidermidis*	19/EPI0130	1		35	A	≤0.12	**>16**	≤0.016	**>32**	**>4**	**1**	**64**	32	≤0.5	≤1	**8**	**>32**	**>8**	**4**	4	≤1	≤0.5	256
**B**	*S. aureus*	18/EPI2714		1	97	A	≤0.12	≤0.5	≤0.016	8	≤0.5	**>2**	8	≤4	≤0.5	≤1	≤1	≤2	0.5	0.5	**>16**	2	≤0.5	≤64
	*S. aureus*	18/EPI2723		1	5	A	≤0.12	≤0.5	≤0.016	8	≤0.5	**>2**	8	≤4	1	≤1	≤1	**>32**	0.5	≤0.25	**>16**	2	≤0.5	≤64
	*S. pseud-intermedius*	18/EPI2623	16	1	551	A	**>4**	**>16**	0.03	**>32**	≤0.5	**>2**	8	**>64**	1	≤1	**8**	**>32**	**>8**	**>8**	**8**	≤1	≤0.5	**>512**
	*S. equorum*	19/EPI0070	1			A	0.5	**>16**	≤0.016	**>32**	≤0.5	**>2**	8	**>64**	1	2	**8**	**>32**	**8**	0.5	**>16**	2	≤0.5	≤64
	*M. caseolyticus*	19/EPI0068	1		39	D	**>4**	**16**	≤0.016	32	**4**	**>2**	**64**	≤4	≤0.5	≤1	≤1	≤2	**>8**	≤0.25	**16**	≤1	≤0.5	≤64
**C**	*S. aureus*	18/EPI2724	1	1	225	A	**>4**	≤0.5	≤0.016	≤4	≤0.5	**>2**	**16**	**64**	1	≤1	≤1	≤2	**>8**	**>8**	**>16**	2	≤0.5	≤64
	*S. pseud-intermedius*	18/EPI2652	1		1339	A	**>4**	**>16**	≤0.016	**>32**	≤0.5	**>2**	≤4	**>64**	≤0.5	≤1	**16**	**>32**	**>8**	**>8**	4	≤1	≤0.5	**>512**
	*S. haemolyticus*	19/EPI0101	11		9	A	**4**	**>16**	≤0.016	≤4	≤0.5	**>2**	≤4	**>64**	1	≤1	≤1	≤2	**>8**	**2**	**8**	≤1	≤0.5	**>512**
	*S. haemolyticus*	19/EPI0069	2		9	A	**4**	**>16**	0.03	≤4	≤0.5	**2**	≤4	**>64**	1	≤1	≤1	≤2	**>8**	**2**	**8**	≤1	≤0.5	**>512**
	*S. epidermidis*	19/EPI0136	1		568	A	**4**	≤0.5	≤0.016	≤4	**>4**	**0.25**	≤4	≤4	1	≤1	≤1	≤2	≤0.25	≤0.25	**8**	≤1	≤0.5	**>512**
	*S. epidermidis*	19/EPI0109	1		910	A	≤0.12	≤0.5	≤0.016	≤4	≤0.5	**>2**	≤4	≤4	≤0.5	2	≤1	≤2	**>8**	≤0.25	**8**	≤1	≤0.5	≤64
	*S. epidermidis*	19/EPI0108	1		88	A	**>4**	1	>0.5	**>32**	**>4**	**2**	8	**>64**	**4**	≤1	**16**	**16**	0.5	1	**>16**	2	**256**	**>512**
**E**	*S. aureus*	19/EPI0122	1		22	A	≤0.12	≤0.5	≤0.016	≤4	≤0.5	**>2**	8	≤4	≤0.5	≤1	≤1	≤2	≤0.25	**>8**	**>16**	2	≤0.5	≤64
	*S. haemolyticus*	19/EPI0116	1		30	A	**>4**	**>16**	≤0.016	≤4	**>4**	**>2**	≤4	32	≤0.5	≤1	**4**	**>32**	**>8**	**8**	**8**	≤1	≤0.5	256
	*S. haemolyticus*	19/EPI0119	2		42	A	**>4**	**>16**	≤0.016	**>32**	**>4**	**>2**	8	**>64**	1	2	**>16**	**>32**	**>8**	**>8**	**>16**	≤1	≤0.5	**>512**
	*S. haemolyticus*	19/EPI0126	1		69	A	≤0.12	1	≤0.016	**>32**	≤0.5	**>2**	≤4	**>64**	≤0.5	2	**>16**	≤2	**>8**	**>8**	**>16**	≤1	≤0.5	**>512**
	*S. haemolyticus*	19/EPI0121	1		69	A	≤0.12	**8**	≤0.016	**>32**	**>4**	**>2**	≤4	**>64**	≤0.5	2	**>16**	≤2	**>8**	**>8**	**>16**	≤1	≤0.5	**>512**
	*M. caseolyticus*	19/EPI0120	4		38	D	≤0.12	**>16**	≤0.016	**>32**	≤0.5	**>2**	≤4	≤4	1	≤1	≤1	≤2	≤0.25	≤0.25	**>16**	≤1	1	≤64
	*M. caseolyticus*	19/EPI0117	1		35	D	≤0.12	≤0.5	≤0.016	≤4	≤0.5	**>2**	≤4	≤4	1	≤1	≤1	4	**>8**	≤0.25	**>16**	≤1	1	≤64
	*M. canis*	19/EPI0114	1		69	B	**4**	≤0.5	> 0.5	32	≤0.5	**>2**	≤4	32	2	≤1	**4**	≤2	0.5	0.5	**>16**	≤1	1	**>512**
	*M. canis*	19/EPI0118	1		71	B-D	0.25	≤0.5	≤0.016	≤4	**2**	**>2**	≤4	≤4	1	≤1	≤1	≤2	≤0.25	≤0.25	**16**	≤1	1	≤64
	*M. canis*	19/EPI0123	1		72	D	0.25	≤0.5	≤0.016	≤4	≤0.5	**2**	≤4	≤4	1	≤1	≤1	≤2	≤0.25	≤0.25	**>16**	≤1	1	≤64
**F**	*S. haemolyticus*	19/EPI0131	1		9	A	**4**	≤0.5	0.03	≤4	**4**	**>2**	≤4	32	**4**	≤1	**4**	≤2	≤0.25	1	**>16**	≤1	≤0.5	**>512**
	*S. epidermidis*	19/EPI0138	1		88	A	≤0.12	1	≤0.016	**>32**	≤0.5	**>2**	≤4	≤4	≤0.5	≤1	≤1	**16**	≤0.25	≤0.25	**8**	≤1	≤0.5	256
**G**	*S. haemolyticus*	19/EPI0073	3		49	A	≤0.12	**>16**	≤0.016	≤4	≤0.5	**>2**	≤4	**64**	≤0.5	≤1	**4**	>32	≤0.25	**>8**	**16**	≤1	≤0.5	256

^a^For the five *S. epidermidis* isolates from employees, no data on antibiotic resistance profile or strain typing was available. ^b^Sequence types of the coagulase-negative staphylococci were determined for the representative strains. ^c^MIC in bold indicates resistance. The resistance breakpoints presented are those for *Staphylococcus* spp. from the European Committee on Antimicrobial Susceptibility Testing [32] except for KAN and SMX for which the breakpoints from the Clinical and Laboratory Standards Institute [33] were used. ^d^No breakpoint was available for STR; an MIC > 32 was tentatively used as resistance breakpoint. ^e^EUCAST breakpoints for vancomycin: for *S. aureus* > 2, for coagulase-negative staphylococci > 4. *Abbreviations: Env* environment, *Empl* employee, *ST* sequence type, *CLI* clindamycin, *TET* tetracycline, *RIF* rifampicin, *STR* streptomycin, *FUS* fusidic acid; PEN, penicillin, *CHL* chloramphenicol, *KAN* kanamycin, *SYN* synercid, *VAN* vancomycin, *GEN* gentamicin, *TMP* trimethoprim, *ERY* erythromycin, *CIP* ciprofloxacin, *FOX* cefoxitin, *LZD* linezolid, *MUP* mupirocin, *SMX* sulfamethoxazole, *S. Staphylococcus, M. Macrococcus*

**Table 2:** Characteristics and antibiotic resistance profile of the Gram–negative multidrug-resistant organisms

Insti-tution	Species	Represen-tative strain	Origin (n=)		ST	β-lactamase genes	Antibiotic resistance profile^**a**^													
							SMX (≥512)	TMP (>4)	CIP (>0.5)	TET (≥16)	MERO (>8) (ESBL screen >0.125)	AZI NA^**b**^	NAL (≥32)	FOT (>2) (ESBL screen >1)	CHL (>8)	TGC (>2)	TAZ (>4) (ESBL screen >1)	COL (>2)	AMP (>8)	GEN (>4)
			Env	Empl																
**A**	*E. coli*	M_054		1	73		32	≤0.25	≤0.015	≤2	≤0.03	4	≤4	≤0.25	≤8	≤0.25	≤0.5	**16**	4	≤0.5
	*E. coli*	M_042		1	543		32	≤0.25	≤0.015	≤2	≤0.03	≤2	≤4	≤0.25	≤8	≤0.25	≤0.5	**16**	4	2
	*E. coli*	M_064		1	2640		32	≤0.25	≤0.015	≤2	≤0.03	4	≤4	≤0.25	≤8	≤0.25	≤0.5	**8**	2	≤0.5
	*E. coli*	M_054		1	1193	CTX-M-15	16	≤0.25	**>8**	≤2	≤0.03	4	**>128**	**>4**	≤8	≤0.25	**4**	≤1	**>64**	**16**
	*E. coli*	M_050		1	167	NDM-5/ CMY-2/TEM-30	**>1024**	**>32**	**>8**	**>64**	**>16**	**>64**	**>128**	**>4**	**>128**	≤0.25	**>8**	≤1	**>64**	**32**
	*E. coli*	M_018		1	131	CTX-M-15	32	0.5	0.25	4	≤0.03	4	**>128**	**>4**	≤8	≤0.25	**>8**	≤1	**>64**	≤0.5
	*E. coli*	M_104		1	95	CTX-M-55	32	0.5	≤0.015	≤2	≤0.03	4	≤4	**>4**	≤8	≤0.25	**8**	≤1	**>64**	≤0.5
	*E. cloacae*	19/EPI0154	1				**>1024**	0.5	0.5	**>64**	**0.25**	32	**32**	1	≤8	0.5	1	≤1	**>64**	4
**B**	*K. pneumoniae*	19/EPI0077	18		11	OXA-48/ DHA-1	**>1024**	4	**>8**	4	**1**	**>64**	**>128**	**>4**	**32**	0.5	**>8**	≤1	**>64**	4
	*E. coli*	19/EPI0083	1		410	OXA-181/ CMY-42	16	2	**>8**	**>64**	**0.5**	16	**>128**	**>4**	**32**	1	**>8**	≤1	**>64**	≤0.5
	*E. coli*	MB_042		1	410	OXA-181/ CMY-42	16	1	**>8**	4	**0.5**	8	**>128**	**>4**	≤8	≤0.25	**>8**	≤1	**>64**	≤0.5
	*E. coli*	19/EPI0060	1		4038	OXA-48	**>1024**	**>32**	**>8**	**>64**	**0.5**	16	**>128**	**>4**	**16**	1	**>8**	≤1	**>64**	≤0.5
	*E. coli*	MB_091		1	538		32	≤0.25	≤0.015	≤2	≤0.03	≤2	≤4	≤0.25	≤8	≤0.25	≤0.5	**>16**	4	≤0.5
	*E. coli*	MB_066		1	95		16	≤0.25	≤0.015	≤2	≤0.03	≤2	≤4	≤0.25	≤8	≤0.25	≤0.5	**8**	2	≤0.5
	*E. coli*	MB_100		1	335		16	≤0.25	≤0.015	≤2	≤0.03	4	≤4	≤0.25	≤8	≤0.25	≤0.5	**4**	2	≤0.5
	*E. coli*	MB_003		1	1730	CTX-M-1	**>1024**	**>32**	≤0.015	≤2	≤0.03	8	≤4	**>4**	**>128**	≤0.25	1	≤1	**>64**	**>32**
	*E. coli*	MB_074		1	131	CTX-M-27	**>1024**	≤0.25	**>8**	**64**	≤0.03	≤2	**>128**	**>4**	≤8	≤0.25	**4**	≤1	**>64**	1
	*E. coli*	MB_073		1	131	CTX-M-14	32	≤0.25	≤0.015	≤2	0.06	8	≤4	**>4**	≤8	≤0.25	**2**	≤1	**>64**	≤0.5
	*E. cloacae*	19/EPI0060	1			OXA-48	≤8	1	≤0.015	≤2	**0.5**	16	≤4	**2**	≤8	≤0.25	≤0.5	≤1	**>64**	≤0.5
	*E. cloacae*	19/EPI0059	1				**>1024**	**>32**	**2**	**>64**	0.12	8	**>128**	**>4**	**>128**	≤0.25	**>8**	≤1	**>64**	4
**C**	*E. coli*	MC_022		1	69		32	≤0.25	≤0.015	< 2	≤0.03	4	≤4	≤0.25	≤8	≤0.25	≤0.5	**8**	4	≤0.5
**E**	*E. coli*	19/EPI0155	1			OXA-48	**>1024**	**>32**	**>8**	**>64**	**0.25**	8	**>128**	**>4**	**>128**	≤0.25	**>8**	≤1	**>64**	≤0.5

^a^MIC in bold indicates resistance. The resistance breakpoints presented are those for *E. coli* from the European Committee on Antimicrobial Susceptibility Testing [32] except for NAL, SMX and TET for which the breakpoints from the Clinical and Laboratory Standards Institute [33] were used. ^b^NA, not available; no breakpoints were available for *E. coli* for azithromycin; an MIC > 64 was tentatively used as resistance breakpoint. *Abbreviations: Env* environment; *Empl* employee, *ST* sequence type, *SMX* sulfamethoxazole, *TMP* trimethoprim, *CIP* ciprofloxacin, *TET* tetracycline, *MERO* meropenem, *AZI* azithromycin, *NAL* nalidixic acid, *FOT* cefotaxime, *CHL* chloramphenicol, *TGC* tigecycline, *TAZ* ceftazidime, *COL* colistin, *AMP* ampicillin, *GEN* gentamicin, *E. coli Escherichia coli*, *E. cloacae Enterobacter cloacae*, *K. pneumoniae Klebsiella pneumoniae*

### Data analysis

Data from employees were collected and managed using REDCap (Research Electronic Data Capture) hosted at the University of Bern, Switzerland [39, 40]. Statistical analysis was performed using SPSS version 24.0.0. (IBM, Zurich, Switzerland) and the freely available software program R version 3.2.0 [41]. A total of 26 categorical parameters were statistically analyzed for association with carriage of Gram-positive or Gram-negative MDROs in employees using the Chi square and Fisher’s exact test (for expected frequencies < 5). *P*-values < 0.05 were considered statistically significant.

## Results

### IPC audit scores

Five small animal clinics and two first opinion practices from Switzerland participated in the study (Additional file 1). Of a maximum total IPC score of 68, the clinics/practices reached a median (range) total IPC score of 33 (19–55) (Table 3). Major IPC deficits in the institutions were absence of written IPC manuals (4/7 institutions), absence of regular IPC audits (7/7 institutions), absence of written and updated protocols for cleaning and disinfection (4/7 institutions), absence of written protocols for quarantine measures (6/7 institutions) and no definition and flagging of patients with MDROs (5/7 institutions, Additional file 4).

**Table 3:** Infection prevention and control scores of the seven small animal clinics/practices

Area of IPC^**a**^	Maximum score	Institution							Sum per area for the evaluated institutions	Maximum sum per area for seven institutions
		Clinic A	Clinic B	Clinic C	Clinic D	Clinic E	Practice F	Practice G		
**IPC management**	6	4	2	4	2	0	0	2	14	42
**Staff education**	6	5	4	3	5	0	0	0	17	42
**Cleaning / disinfection**	6	5	2	2	6	3	3	4	25	42
**Quarantine measures**	6	6	1	2	3	1	3	0	16	42
**Guidelines for patients with MDROs**	4	3	3	1	1	0	1	0	9	28
**Hand hygiene**	8	7	3	5	8	2	6	3	34	56
**Personal hygiene**	12	9	7	5	9	3	6	2	41	84
**Protection of employees**	4	2	3	2	1	3	3	2	16	28
**Protective clothing**	6	6	4	4	4	3	4	0	25	42
**Medication**	6	4	2	5	6	3	3	6	29	42
**Guidelines/restrictions for antimicrobial use**	4	4	4	0	3	1	4	1	17	28
**Total score**	**68**	**55**	**35**	**33**	**48**	**19**	**33**	**20**	**243**	**476**
**% of maximum score**	**100%**	**81%**	**52%**	**49%**	**71%**	**28%**	**49%**	**29%**	**51%**	**100%**

^a^The total score per IPC area is shown. For detailed scoring see Additional file 4

*Abbreviations: IPC* Infection prevention and control, *MDROs* multidrug resistant organisms

### Environmental contamination with MDROs

Results for environmental contamination with MDROs in the Clinics A–C and Clinics/Practices D–G are shown in Tables 4 and 5, respectively. Details on species, strains and antibiotic resistance profiles of the Gram-positive and Gram-negative isolates are given in Tables 1 and 2, respectively. Overall, MDROs were found in median (range) 8.2% (0–33.3%) of the environmental sampling sites in the seven institutions; consultation rooms, ICUs and utensils were most commonly contaminated. In the additional samples collected in Clinic B, 22% tested positive for CP *K. pneumoniae* (ST11, *bla*_OXA-48_, *n* = 13); samples from the emergency room and ICU accounted for 12/13 positive samples. The results of the IPC scoring in the clinics were in accordance with the extent of environmental contamination. Clinics B, C and E with scores of 35, 33 and 19, respectively, showed most extensive environmental contamination, with 30, 32 and 33% of the sampling sites testing positive for MDROs, respectively (Tables 3, 4 and 5).

**Table 4:** Results of environmental examinations for multidrug-resistant organisms in Clinics A–C

Area	Clinic A^**a**^			Clinic B^**a**^					Clinic C^**a**^	
	No. of sampling sites^**b**^	MRS	ESBL-PE	No. of sampling sites^**b**^	MRS	ESBL-PE	CPE	*Macrococcus* spp.	No. of sampling sites^**b**^	MRS
Waiting area	3	**1**	0	3	0	0	0	0	3	**1**
Consultation rooms	6	0	0	6	**5**	0	**2**	**1**	3	0
ICU	4	**2**	0	6	**5**	0	**4**	0	6	**4**
Radiology	3	0	0	3	0	0	0	0	1	**1**
Ward	3	0	**1**	4	**1**	0	**2**	0	3	**1**
Quarantine ward	1	0	0	3	0	0	0	0	3	**2**
Pre-OR area	7	**1**	0	6	0	0	0	0	6	**1**
OR	10	0	0	10	0	0	0	0	10	0
Laboratory	4	0	0	4	**1**	0	0	0	3	**1**
Endoscopy	1	0	0	1	0	0	0	0	1	0
Dental room	3	0	0	3	0	0	0	0	2	0
Utensils^c^	9	0	0	9	**3**	0	0	0	8	**5**
Others^d^	7	0	0	8	**2**	**1**	0	0	4	**2**
Total no. of sampling sites	61			66					53	
Total no. (%) of positive sampling sites	**5 (8)**	**4 (7)**	**1 (2)**	**20 (30)**	**17 (26)**	**1 (2)**	**6 (9)**^**e**^	**1 (2)**	**17 (32)**	**17 (32)**^**f**^

^a^ Results for all included multidrug-resistant organisms are shown (Methicillin-resistant staphylococci, ESBL- and carbapenemase-producing *Enterobacterales* and *Macrococcus* spp.); missing rows indicate no positive result for this group of multidrug-resistant organisms. Positive results are shown in bold. Details on species, strain and minimal inhibitory concentrations of the isolates are specified in Tables 1 and 2. ^b^Details on sampling sites are indicated in Additional file 3. ^c^Utensils: transport boxes, transport trolleys, mobile phones / pagers, stethoscopes, thermometers, otoscopes, clippers, ultrasonography devices, scissors / clamps, sharp drops. ^d^Others: elevator, bath, feeding kitchen, lingerie, staff toilet, pneumatic dispatch system. ^e^In two sampling sites, two different isolates were found. ^f^In one sampling site, two different isolates were found. *Abbreviations: MRS* Methicillin-resistant staphylococci, *ESBL-PE* Extended spectrum ß-lactamase-producing *Enterobacterales, CPE* carbapenemase-producing *Enterobacterales, ICU* intensive care unit, *OR* operating room

**Table 5:** Results of environmental examinations for multidrug-resistant organisms in Clinics/Practices D–G

Area	Clinic D^a^		Clinic E^a^				Practice F^a^		Practice G^a^	
	No. of sampling sites^**b**^	All MDROs tested	No. of sampling sites^**b**^	MRS	CPE	*Macrococcus* spp.	No. of sampling sites^**b**^	MRS	No. of sampling sites^**b**^	MRS
Waiting area	2	0	2	**1**	0	0	2	0	2	**1**
Consultation rooms	4	0	5	**1**	**1**	**2**	5	**2**	5	0
Radiology	1	0	2	0	0	**1**	2	0	1	**2**
Ward	4	0	5	**1**	0	**1**	7	0	6	0
Pre-OR area	4	0	4	**1**	0	**1**	4	0	2	0
OR	7	0	4	0	0	0	3	0	5	0
Laboratory	3	0	4	**1**	0	0	5	0	4	0
Office	2	0	1	0	0	0	2	0	0	0
Utensils^c^	8	0	8	**1**	0	**2**	8	0	8	0
Others^d^	3	0	4	0	0	**1**	2	0	4	0
Total no. of sampling sites	38		39				40		37	
Total no. (%) of positive sampling sites	**0 (0)**	**0 (0)**	**13 (33)**	**6 (15)**	**1 (3)**	**8 (21)**	**2 (5)**	**2 (5)**	**3 (8)**	**3 (8)**

^a^ Results for all included MDROs are shown (Methicillin-resistant staphylococci, ESBL- and carbapenemase-producing *Enterobacterales* and *Macrococcus* spp.); missing rows indicate no positive result for this group of MDROs. Positive results are shown in bold. Details on species, strain and minimal inhibitory concentrations of the isolates are specified in Tables 1 and 2. ^b^Details on sampling sites are indicated in Additional file 3. ^c^Utensils: phones, stethoscopes, thermometers, otoscopes, clippers, ultrasonography devices, clamps / scissors, muzzles, dental cleaning devices / utensils. ^d^Others: feeding utensils, lingerie, changing rooms, sterilizer

*Abbreviations: MDROs* multidrug resistant organisms, *MRS* Methicillin-resistant staphylococci, *CPE* carbapenemase-producing *Enterobacterales, OR* operating room

In Clinic B, the environment was highly contaminated with MRSP (ST551, *mecA*) and CP *K. pneumoniae* (ST11, *bla*_OXA-48_); CP *E. coli* (ST410, *bla*_OXA-181_; ST4038, *bla*_OXA-48_) were found in single sampling sites (Tables 1 and 2). All 16 MRSP (ST551, *mecA*) isolates showed similar resistance profiles (Table 1). The total 18 CP *K. pneumoniae* (ST11, *bla*_OXA-48_) isolates from the two sample collections in Clinic B also shared similar resistance profiles (Table 2) and showed the same REP-PCR profile (data not shown). In Clinic C, the environment was highly contaminated with MRCoNS (Table 1). Thirteen MR *Staphylococcus haemolyticus* isolates (ST9, *mecA*) were detected which showed similar resistance profiles (Table 1); 11 and 2 *S. haemolyticus* isolates showed the same ERIC-PCR profiles, respectively (data not shown). In Clinic E, a diverse population of MDROs was found, predominated by MR *Macrococcus* spp. (ST69, *mecB*; ST71, *mecB–D;* ST35, ST38, ST72*, mecD*) and *S. haemolyticus* (ST30, ST42, ST69, *mecA*) isolates (Table 1); a single CP *E. coli* (*bla*_OXA-48_) isolate was detected in a consultation room (Table 2). The MR *S. haemolyticus* isolates in Clinic E showed no clonal relationship in ERIC-PCR (data not shown) and different antibiotic resistance profiles (Table 1).

### MDRO colonization of employees

A total of 109 employees of Clinics A–C and Practice G provided samples (99 nasal swabs and 108 fecal samples) and 108 employees completed the questionnaire. Results for MDRO carriage in employees are shown in Table 6, details on the detected Gram-positive and Gram-negative isolates are shown in Tables 1 and 2, respectively.

**Table 6:** Colonization with multidrug-resistant organisms of employees of Clinics A–C and Practice G

Institution	Total no. of sampled employees (nasal swabs / fecal samples)	No. of sampled			No. (%)^**a**^ of employees positive for						Total no. (%)^**b**^ of positive employees
		Veterinarians	Nurses	Others/unknown^**c**^	MRSA	MRSP	MRCoNS	ESBL-PE	CPE	CRE	
**Clinic A**	38 (37/37)	19	12	7	4 (11)	0 (0)	2 (5)	3 (8)	1 (3)	3 (8)	12 (32)^d^
**Clinic B**	46 (38/46)	22	13	11	2 (5)	1 (3)	3 (8)	3 (7)	1 (2)	3 (7)	13 (28)
**Clinic C**	21 (20/21)	14	6	1	1 (5)	0 (0)	0 (0)	0 (0)	0 (0)	1 (5)	2 (10)
**Practice G**	4 (4/4)	1	3	0	0 (0)	0 (0)	0 (0)	0 (0)	0 (0)	0 (0)	0 (0)
**Total**	**109 (99/108)**	**56**	**34**	**19**	**7 (7)**	**1 (1)**	**5 (5)**	**6 (6)**	**2 (2)**	**7 (7)**	**27 (25)**^d^

^a^ Percentage related to total number of nasal swabs or fecal samples available from the according institution. ^b^ Percentage related to number of sampled employees. ^c^ Profession not known for 5 participating employees. ^d^One employee tested positive for both an ESBL-producing and colistin-resistant *Enterobacterales*

*Abbreviations: MRSA* methicillin-resistant *Staphylococcus aureus, MRSP methicillin-resistant Staphylococcus pseudintermedius, MRCoNS* methicillin-resistant coagulase-negative staphylococci, *ESBL-PE* extended-spectrum β-lactamase-producing *Enterobacterales*, *CPE* carbapenemase-producing *Enterobacterales*, *CRE* colistin-resistant *Enterobacterales*

A total of 13 (13%) of the employees carried MR *Staphylococcus* spp., comprising 7 genetically diverse MRSA (ST398, ST7, ST45, ST97, ST5, ST225; *mecA*), one MRSP (ST551, *mecA*) and five MR *S. epidermidis* isolates. MR *Staphylococcus* spp. of the same sequence types and with similar AMR profiles were found in both employees and the environment in Clinic B (MRSP ST551) and Clinic C (MRSA ST225) (Table 1); no data on antibiotic resistance profiles and sequence typing were available for the MR *S. epidermidis* isolates. Carriage of MR *Staphylococcus* spp. was associated with the presence of an actual disease (*p* = 0.036) and contact to horses during work (*p* = 0.049). Actual diseases listed by employees with carriage of MR *Staphylococcus* spp. were: cystitis (*n* = 1), chronic sinusitis (*n* = 2) and Morbus Basedow (*n* = 2).

A total of 6 (6%) and 7 (7%) employees tested positive for ESBL-producing and COL-R *Enterobacterales*, respectively. There was no evidence for clonal relationship of these isolates based on sequence type, antibiotic resistance profiles (Table 2) and REP-PCR (data not shown). Three employees were colonized with *E. coli* ST131 (*bla*_CTX-M-15_, *bla*_CTX-M-27_, *bla*_CTX-M-14_, Table 2). Two staff members were found to be carriers of CP *E. coli*; details of these isolates have been published [42]. A staff member in Clinic A carried a CP *E. coli* (ST167, *bla*_NDM-5,_*bla*_CMY-2,_*bla*_TEM-30_). In Clinic B, a staff member showed fecal carriage of a CP *E. coli* (ST410, *bla*_OXA-181,_*bla*_CMY-42_) isolate that was closely related to an isolate from the environment [42]. A significant association of carriage of Gram-negative MDROs was found for living in an urban environment (*p* = 0.026) and lack of outdoor activities (*p* = 0.017).

## Discussion

This study documents variable IPC standards in companion animal clinics and practices in Switzerland and extensive contamination with MDROs in institutions with low IPC standards. Worryingly, CP *Enterobacterales* were detected in the environment of two companion animal clinics, one of them showing extensive contamination with both CP *K. pneumoniae* (ST11, *bla*_OXA-48,_*bla*_DHA-1_) and MRSP (ST551, *mecA*). The results of the study suggest that insufficient IPC standards in companion animal clinics are associated with environmental contamination with MDROs and may increase the risk of potential transfer of MDR bacteria between the clinical environment, patients and employees. In this context, two employees in this study were found to be colonized with CP *E. coli* closely related to environmental or patient-derived isolates [43], and two staff members carried MRSA and MRSP of the same sequence type as found in the environment in the according institution, respectively; some of these results have been published [11, 42]. CPE have been classified as an “urgent” public health threat by the US Centers for Disease Control and Prevention because nosocomial infections in humans have been associated with a case fatality rate of up to 50% [44, 45]. Carbapenems are used as a last-resort therapy for invasive Gram-negative infections in humans [46]. Although not licensed for use in animals, they have been reported to be used occasionally in dogs and cats [5]. Interestingly, carbapenems have never been used in patients of Clinic B, suggesting that the development and clonal spread of the CPE in the environment of this clinic was not driven by selection pressure exerted by carbapenems [11].

Several deficits in IPC could have contributed to the extensive spread of MRSP and CPE in Clinic B. The clinic had no written protocols on cleaning and disinfection in place. Disposable gloves were worn at every patient contact and routine hand disinfection was inconsistently performed. Utensils such as scissors or clippers were personalized to the staff members and carried in personal bum bags. This carries the risk of contamination of the utensils within the bags which could than act as vehicles to transfer MDROs between patients. In Switzerland, like in many other European countries, no legislation regulates the IPC standards in small animal veterinary clinics and practices. Furthermore, education of veterinary students and practicing veterinarians in IPC is hampered by a lack of IPC specialists and training programs in companion animal medicine [47]. Immediately after the discovery of the outbreak in Clinic B, a comprehensive IPC concept was implemented with the support of IPC professionals, hand disinfection dispensers were placed throughout the clinic and hand hygiene training has been initiated for all employees.

Overall, the IPC standards found in the companion animal clinics in Switzerland were variable. The deficits in IPC were illustrated by i.e. the absence of written IPC manuals in 4/7 institutions, no staff education on any IPC-related topics in 3/7 institutions, no definition and flagging of MDRO patients in 5/7 institutions, and no written and updated protocols on quarantine measures in 6/7 institutions. Clinic E with the lowest total IPC score had no IPC management in place at all, no hand sanitizers and dispensers were available throughout the clinic and no guidelines for the handling of patients with MDROs were established.

Antimicrobial use is thought to be a major driving force towards antimicrobial resistance [3]. The IPC audits in this study assessed whether specified guidelines on the use and dosing of antimicrobials were available for all employees involved in prescription and application of antimicrobials, and whether use of antibiotics of last resort were restricted and limitations communicated and known to all employees; these aspects were completely fulfilled by 4/7 and 3/7 institutions, respectively. However, overall consumption of antimicrobials was not assessed because this data was not collected and monitored by the investigated institutions. An information system for antimicrobials (IS ABV) to monitor their consumption in all veterinary clinics and practices in Switzerland was introduced by the Swiss Federal Food Safety and Veterinary Office in October 2019 [48], after conclusion of this study, but reliable data from the system is not yet available.

Four institutions (A–D) included in the present study were recently evaluated for antimicrobial prescription habits in dogs and cats in two unrelated studies [49, 50]. In these investigations, the frequency of prescription and adherence to prudent use guidelines for three disease complexes in cats and four disease complexes in dogs were evaluated between January and December 2016. There was no difference in overall prescription rate among Clinics A–D for the evaluated cases (247 feline and 431 canine cases; BW and SS, personal communication). We therefore assume that overall antibiotic use was not a major contributor to the differences in environmental contamination with MDROs observed in Clinics A–D.

Whether the extensive environmental contamination poses a nosocomial infection risk for the patients in these institutions cannot be answered from this study. None of the evaluated institutions had an active surveillance of health care-associated infections in place. However, retrospective investigations to assess the role of CPE in nosocomial infections in Clinic B are underway.

Areas with high patient traffic, such as consultation rooms and ICUs, and utensils were most commonly contaminated with MDROs. High rates of contamination with MR staphylococci in high traffic areas within a veterinary hospital have been documented [51]. The clonal relationship of the MDROs in the environment of Clinics B and C also suggests a spread from a common source in these institutions. Practices F and G on the other hand reached rather low IPC scores in fact, however, environmental contamination was low in both practices. This could indicate that first opinion practices in contrast to large referral clinics are less critical in the development and spread of MDROs. Studies in human medicine suggest that the influx of resistant pathogens varies significantly in hospitals of different size, location and patient groups [52]. Therefore, a large clinic could be at higher risk for the introduction of MDR pathogens than first opinion practices [2]. Since the number of first opinion practices included in this study was low, no conclusions can yet be drawn and future studies should address the role of first opinion practices in the development and spread of MDROs.

The prevalence of colonization with ESBL-producing or COL-R *Enterobacterales* in veterinary employees in this study was comparable to the colonization rate in the general population in Switzerland [53, 54]. Three employees from Clinics A and B were found to be colonized with *E. coli* ST131 (*bla*_CTX-M-15_, *bla*_CTX-M-27_, *bla*_CTX-M-14_). *E. coli* ST131 is one of the most important globally disseminated bacterial lineage and causes severe hospital-acquired and community-onset MDR infections in humans [55]. Infections with *E. coli* ST131 have, amongst others, also been reported in companion animals [56]. Of note, an unrelated study investigated clinical samples originating from canine and feline patients from Clinic A and found that the prevalence of ESBL-producing uropathogenic *E. coli* ST131 had increased between 2010 and 2012 from 0 to 1.5%, and included *E. coli* ST131 (*bla*_CTX-M-15_ and *bla*_CTX-M-27_) [57].

The prevalence of colonization with MRSA in veterinary employees in this study was higher than reported for the general population in Switzerland (7% vs 1.5%) [58]. Variable MRSA sequence types were detected in the employees, including healthcare-associated (ST225, ST5, ST45, ST7), livestock-associated (ST398) and community-acquired MRSA (ST97). Veterinarians have an occupational risk for acquisition of MRSA [59]. Contact with horses has been reported to be a risk factor for acquiring MRSA ST398 [60] and was also found to be associated with colonization with MR staphylococci in this study. Carriage of MRSP has been well documented in veterinarians and owners with contact to MRSP infected animals [16, 17, 21, 61, 62]. MRSP ST551 has so far only occasionally been reported in companion animals in Europe, but was found to have become the dominating MRSP lineage in dogs in Poland, but emerged not before 2015 [63]. An occupational acquisition of MRSP ST551 by the employee in Clinic B due to extensive environmental contamination with this clone seems most likely.

Environmental detection of macrococci was common in this study, especially in Clinic E. The identified sequence types were highly variable, which was recently also found for macrococci isolated from carriage and infection sites of dogs [27]. The macrococci sequence types found in the environment did not match the lineages recently documented in dogs in Switzerland [27]. The clinical significance of macrococci in companion animals and the impact of environmental contamination with macrococci has not yet been resolved and needs further investigations.

## Conclusions

The present study documents variable IPC standards in companion animal clinics and practices in Switzerland. Low IPC standards in clinics were associated with extensive environmental MDRO contamination. The detection of an MRSP and CP *Enterobacterales* outbreak in one clinic and of closely related MDRO isolates in employees, patients and the environment in several clinics indicate that insufficient IPC standards in companion animal clinics may pose a public health risk. The results suggest that companion animal clinics can significantly contribute to the development and dissemination of MDROs. Proper IPC standards in small animal veterinary clinics and educational programs in IPC for veterinary students and practitioners should therefore urgently be promoted.

## Supplementary information

**Additional file 1.** Characteristics of the seven small animal veterinary clinics and practices included in the done study.

**Additional file 2.** Criteria used for infection prevention and control (IPC) scoring in the seven companion animal institutions and as evaluated during one-day IPC audits. Each IPC topic was scored as follows: all criteria fulfilled = score 2; part of the criteria fulfilled = score 1; no criteria fulfilled = score 0. A score per IPC area and a total score was calculated. The results of the IPC scoring of the participating institutions are given in Table 3 and Additional file 4.

**Additional file 3.** Environmental sampling sites in Clinics A–C and Clinics/Practices D–G.

**Additional file 4.** Detailed infection prevention and control scoring for Clinics/Practices A–G.

## Data Availability

Thirteen whole genome sequences have been deposited on BioProject PRJNA587632 in GenBank. The datasets used and analyzed during this study are available from the corresponding author on reasonable request.

## Abbreviations

AMR: Antimicrobial resistance
CLSI: Clinical and Laboratory Standards Institute
COL-R: Colistin-resistant
CP: Carbapenemase-producing
CPE: Carbapenemase-producing *Enterobacterales*
EUCAST: European Committee on Antimicrobial Susceptibility Testing
ESBL: Extended-spectrum β-lactamase
IPC: Infection prevention and control
ICU: Intensive care unit
MALDI-TOF MS: Matrix-Assisted-Laser-Desorption/Ionization-Time-Of-Flight-Mass-Spectrometry
MR: Methicillin-resistant
MRCoNS: Methicillin-resistant coagulase-negative staphylococci
MRSA: Methicillin-resistant *Staphylococcus aureus*
MRSP: Methicillin-resistant *Staphylococcus pseudintermedius*
MICs: Minimal inhibitory concentrations
MDR: Multidrug-resistant
MDROs: Multidrug-resistant organisms
