# Supplementary material for: Poor infection prevention and control standards are associated with environmental contamination with carbapenemase-producing Enterobacterales and other multidrug-resistant bacteria in Swiss companion animal clinics
Source: Antimicrob Resist Infect Control. 2020 Jun 23;9:93. doi: 10.1186/s13756-020-00742-5 (PMC7310346; doi:10.1186/s13756-020-00742-5)
Supplement: Supplementary file 1 — Additional file 1. Characteristics of the seven small animal veterinary clinics and practices included in the done study. [file 13756_2020_742_MOESM1_ESM.pdf]

Additional file 1. Characteristics of the seven small animal veterinary clinics and practices included in the study.

Institution	Clinic A	Clinic B	Clinic C	Clinic D	Clinic E	Practice F	Practice G
**Approximate no. of cases per year**	8000–10000	6000–8000	> 10000	6000–8000	4000–6000	6000-8000	2000–4000
**No. of staff**	224	110	82	20	11	10	5
**Primary opinion cases**	Yes	Yes	Yes	Yes	Yes	Yes	Yes
**Referred cases**	Yes	Yes	Yes	Yes	Yes	No	No
**24/7 emergency service**	Yes	Yes	Yes	No	No	No	No
**Intensive care unit**	Yes	Yes	Yes	No	No	No	No
**Geographic region in Switzerland**	Central	Central	Central	Western	Southern	Eastern	Western
