# Supplementary material for: Poor infection prevention and control standards are associated with environmental contamination with carbapenemase-producing Enterobacterales and other multidrug-resistant bacteria in Swiss companion animal clinics
Source: Antimicrob Resist Infect Control. 2020 Jun 23;9:93. doi: 10.1186/s13756-020-00742-5 (PMC7310346; doi:10.1186/s13756-020-00742-5)
Supplement: Supplementary file 2 — Additional file 2. Criteria used for infection prevention and control (IPC) scoring in the seven companion animal institutions and as evaluated during one-day IPC audits. Each IPC topic was scored as follows: all criteria fulfilled = score 2; part of the criteria fulfilled = score 1; no criteria fulfilled = score 0. A score per IPC area and a total score was calculated. The results of the IPC scoring of the participating institutions are given in Table 3 and Additional file 4. [file 13756_2020_742_MOESM2_ESM.pdf]

Additional file 2. Criteria used for infection prevention and control (IPC) scoring in the seven companion animal institutions and as evaluated during one-day IPC audits. Each IPC topic was scored as follows: all criteria fulfilled= score 2; part of the criteria fulfilled= score 1; no criteria fulfilled= score 0. A score per IPC area and a total score was calculated. The results of the IPC scoring of the participating institutions are given in Table 3 and Additional file 4.

IPC area	IPC topic	Criteria for assessment
**IPC management**	Hygiene manual	Written IPC manual containing the relevant areas of IPC and provided in written/online form to all employees
	Designated IPC team/person	A defined person or group of people (in large clinics) responsible for IPC implementation, control and teaching that has the according knowledge; in case of IPC groups: regular meetings take place
	Regular audits	IPC audits in the clinic/practice by the IPC mandatory/team or an external IPC advisor, taking place on a regular base
**Staff education**	Hand hygiene	Education in hand hygiene for all employees involved in clinical service, hold on a regular base
	General IPC	Education in infection prevention and control for all employees involved in clinical service, hold on a regular base
	Antimicrobial stewardship	Education in antimicrobial stewardship for all employees involved in prescription and application of antimicrobials, hold on a regular base
**Cleaning/disinfection**	Written & updated protocols	Disinfection protocols with type of disinfection, concentrations and residence times defined for different areas/equipment; written checklists to confirm regular cleaning/disinfection in different areas
	Spectrum and application	Licensed disinfection compounds for clinical use with sufficient bactericidal and virucidal spectrum and use in accordance with the manufacturer's instructions; cleaning procedures that allow for removal of organic matter and proper disinfection
	Information dissemination	Written protocols in all areas and available to all people involved in cleaning/disinfection
**Isolation measures**	Structure and work-flow	For clinics: patients with potential contagious diseases physically separated from other patients; isolation ward that allows for adequate patient separation that contains an upstream area with provision of hand sanitizer and protective clothing; utensils and equipment are assigned to each patient and remain in the isolation area until final cleaning/disinfection; no additional material stored within the isolation room<br>For practices: potentially contagious ambulatory patients are summoned after other consultations; patient movement in the practice is restricted

	Information dissemination	Diseases requiring specific isolation/protective measures are specified and the information is available in written form to all employees
	Cleaning / disinfection	Disinfectants also covering parvovirus and fungal spores are used in the isolation areas; cleaning/disinfection procedures are defined and available to all employees
**Guidelines for patients with MDROs**	Definition/designation of MDRO patients	MDROs are defined and patients with MDRO infections are designated
	Protective measures	MDRO-infected patients are physically separated from other patients, patient movement in the institution is restricted, disinfection procedures are defined
**Hand hygiene**	Hand sanitizer	Hand sanitizer in single-use containers and dispensers are available at all hand washing stations; hand washing stations in all consultation and treatment rooms, wards, laboratories and toilets
	Washing lotion	Medical hand washing lotion in single-use containers available at all hand washing stations (see above)
	Skin protection products	Presence of skin care products available at all hand washing stations (see above)
	Disposable towels	Use of single-use disposable towels at all hand washing stations (see above)
**Personal hygiene**	Working clothes	Provision of working clothes (trousers, and top or coat) for all employees
	Hand jewelry and nails	No hand jewelry (watches, rings, bracelets or similar) and no long or artificial nails and nail polish allowed for employees involved in clinical work
	Food consumption	No consumption of food by employees in the patient areas
	Food storage	No food of employees stored within the patient areas/refrigerators
	Personnel changing rooms	Changing rooms available for all employees that allow to separate private and working clothes
	Laundry	Daily change of working clothes specified; working clothes are cleaned by an external company or by an in-house washing machine in the clinic
**Protection of employees**	Vaccinations	Tetanus and rabies vaccination recommended for all employees and costs covered by the clinic/practice
	Measures for pregnant/immunosuppressed employees	Protective measures in case of pregnancy and/or immunosuppression defined and communicated to all employees

**Protective clothing**	Composition	Complete protective equipment including disposable protective coats or overalls, gloves and shoe covers
	Use	Situation requiring protective clothing and correct use of protective clothing specified and information provided in written form to all employees involved in clinical work
	Storage and provision	Clinics: Protective clothing provided in front of the isolation area and with low potential for contamination of the equipment; Practices: stored with low potential for contamination
**Medication**	Preparation	Preparation of medication on a clean and disinfected working area; avoidance of preparation of medication beforehand
	Storage	Storage according to official regulations (e.g. temperature, storage time); in a clean area/fridge; with no contact to food, feed or patient samples
	Dating of open vials	Dating of open vials and consistently done for all medications
**Guidelines and restrictions for antimicrobial use**	Guidelines for antimicrobial use and dosing	Specified guidelines on use and dosing of antimicrobials available for all employees involved in prescription and application of antimicrobials
	Restrictions for critically important antimicrobials	Limited or prohibited use of antibiotics of last resort, restrictions are communicated and known to all employees

Abbreviations: IPC, Infection prevention and control; MDROs, multidrug resistant organisms.
