# Supplementary material for: Poor infection prevention and control standards are associated with environmental contamination with carbapenemase-producing Enterobacterales and other multidrug-resistant bacteria in Swiss companion animal clinics
Source: Antimicrob Resist Infect Control. 2020 Jun 23;9:93. doi: 10.1186/s13756-020-00742-5 (PMC7310346; doi:10.1186/s13756-020-00742-5)
Supplement: Supplementary file 3 — Additional file 3. Environmental sampling sites in Clinics A–C and Clinics/Practices D–G. [file 13756_2020_742_MOESM3_ESM.pdf]

Additional file 3. Environmental sampling sites in Clinics A–C and Clinics/Practices D–G.

**Clinics A–C**			
**Area**	**Area specified**	**Designation**	**Sampling sites**
**Waiting area**		Lady's toilet	Door handles & flushing bottoms
		Men's toilet	Door handles & flushing bottoms
		Balance	Surface & bottoms
**Consultation rooms**	**General examination rooms**	Desktop	Phone, mouse & keyboard
		Examination tables	Surface
		Cupboards	Handles
		Door & light switch	Handles & switches
	**Emergency room**	Examination tables	Surface
		Cupboards	Handles
**ICU**		Desktop	Phone, mouse & keyboard
		Central examination area	Surface
		Oxygen boxes	Surface
		Infusion pumps	Surface
		Animal boxes	Surface
		Collars & leashes	Surface
**Radiology**	**General radiology**	Working area	Examination table, cassette & release bottom
		Desktop	Phone, mouse & keyboard
	**Radiology in OR**		Keyboard & bottoms
**Ward**		Animal boxes for dogs	Surface
		Animal boxes for cats	Surface
		Waste drains	Surface
		Physiotherapy	Mats & pillows
**Quarantine ward**		Animal boxes	Surface

**Clinics/Practices D–G**		
**Area**	**Designation**	**Sampling sites**
**Waiting area**	Rest rooms	Door handles & flushing bottoms
	Balance	Surface & bottoms
**Consultation rooms**	Desktop	Phone, mouse & keyboard
	Examination tables	Surfaces
	Cupboards	Handles
	Door & light switch	Handles & switch
	Working surface	Surface
**Radiology**	Working area	Examination table, cassette & release bottom
	Desktop	Phone, mouse & keyboard
**Ward**	Animal boxes for dogs	Surface
	Animal boxes for cats	Surface
	Infusion pump	Surface
	Heat mat / heating lamp	Surface
	Mobile balance	Surface & bottoms
	Cupboards	Handles
	Working surface	Surface
**Pre-OR area**	Examination tables	Surface
	Anesthetic apparatus	Surface
	Cupboards	Handles
	Working surface	Surface
	Desktop	Phone, mouse & keyboard
**OR**	Table	Surface
	Heat mat / Bear hugger	Surface

		Cupboards	Handles
		Balance	Surface & bottoms
**Pre-OR area**		Examination tables	Surface
		Warming mats / Bear hugger	Surface
		Anesthetic apparatus	Surface
		Animal boxes	Surface
		Cupboards	Handles
	**Office**	Desktop	Phone, mouse & keyboard
		Table & shelfs	Surface
**OR**	**Septic OR**	Table	Surface
		Warming mat / Bear hugger	Surface
		Anesthetic apparatus	Surface
		Surgical threats cartoons	Surface
		Antiseptic bottle	Surface
	**Orthopedic OR**	Table	Surface
		Warming mat / Bear hugger	Surface
		Anesthetic apparatus	Surface
		Surgical threats cartoons	Surface
		Antiseptic bottle	Surface
**Laboratory**		Centrifuge & blood gas device	Bottoms
		Desktop	Phone, mouse & keyboard
		Microscope	Surface
		Door	Handle
**Endoscopy**		Working canal	Entry & exit
**Dental room**		Examination table	Surface
		Anesthetic apparatus	Surface

	Anesthetic apparatus	Surface
	Surgical threats cartoons	Surface
	Antiseptic bottle	Surface
	Working surface	Surface
	Cupboards	Handles
**Laboratory**	Centrifuge & blood gas device	Bottoms
	Desktop	Phone, mouse & keyboard
	Microscope	Surface
	Doors & cupboards	Handles
	Working surface	Surface
**Office**	Desktop	Phone, mouse & keyboard
	Table & shelfs	Surface
**Utensils**	Mobile phones / pagers	Surface
	Stethoscopes	Surface
	Thermometers	Surface
	Otoscopes	Surface
	Clippers	Surface
	Ultrasonography devices	
	Scissors & clamps	Surface
	Muzzles	Surface
	Dental cleaning devices / utensils	Surface
**Others**	Flatware & bowls	Surface
	Lingerie	Washing machine & dryer, surface
	Changing room	Sheds, cabinets
	Sterilizer	Inner surface
	Sterilizer	Handles, buttons

		Dental cleaning device	Surface
**Utensils**		Transport boxes	Surface
		Transport trolleys	Surface
		Mobile phones / pagers	Surface
		Stethoscopes	Surface
		Thermometers	Surface
		Otoscopes	Surface
		Clippers	Surface
		Ultrasonography device	Surface
		Scissors & clamps	Surface
		Sharp drops	Surface
**Others**	**Elevator**	Bottoms	Surface
	**Bath**	Working area	Surface & handles
	**Feeding kitchen**	Working area	Surface & handles
	**Feeding kitchen**	Flatware & bowls	Surface
	**Lingerie**	Washing machine & dryer	Surface
	**Staff toilet**	Ladies room, door handle & flushing bottoms	Door handles & flushing bottoms
	**Pneumatic dispatch system**	Door handle	Surface
	**Pneumatic dispatch system**	Containers	Surface

Abbreviations: ICU, intensive care unit; OR, operating room.
