# Supplementary material for: Poor infection prevention and control standards are associated with environmental contamination with carbapenemase-producing Enterobacterales and other multidrug-resistant bacteria in Swiss companion animal clinics
Source: Antimicrob Resist Infect Control. 2020 Jun 23;9:93. doi: 10.1186/s13756-020-00742-5 (PMC7310346; doi:10.1186/s13756-020-00742-5)
Supplement: Supplementary file 4 — Additional file 4. Detailed infection prevention and control scoring for Clinics/Practices A–G. [file 13756_2020_742_MOESM4_ESM.pdf]

Additional file 4. Detailed infection prevention and control scoring for Clinics/Practices A–G.

Area	Topic		Institution							Sum per area for the 7 evaluated institutions	Maximum possible sum per area for 7 institutions
			Clinic A	Clinic B	Clinic C	Clinic D	Clinic E	Practice F	Practice G		
**IPC management**	**Hygiene manual**		2	1	2	0	0	0	0	5	14
	**Designated IPC team/person**		2	1	2	2	0	0	2	9	14
	**Regular audits**		0	0	0	0	0	0	0	0	14
		**Sum**	**4**	**2**	**4**	**2**	**0**	**0**	**2**	**14**	**42**
**Staff education**	**Hand hygiene**		2	2	2	2	0	0	0	8	14
	**General IPC**		2	1	1	1	0	0	0	5	14
	**Antimicrobial stewardship**		1	1	0	2	0	0	0	4	14
		**Sum**	**5**	**4**	**3**	**5**	**0**	**0**	**0**	**17**	**42**
**Cleaning/disinfection**	**Written & updated protocols**		1	0	0	2	0	0	1	4	14
	**Spectrum and application**		2	1	2	2	2	2	1	12	14
	**Information dissemination**		2	1	0	2	1	1	2	9	14
		**Sum**	**5**	**2**	**2**	**6**	**3**	**3**	**4**	**25**	**42**
**Quarantine measures**	**Structure and work-flow**		2	1	1	1	0	2	0	7	14
	**Information dissemination**		2	0	0	0	0	0	0	2	14
	**Cleaning / disinfection**		2	0	1	2	1	1	0	7	14
		**Sum**	**6**	**1**	**2**	**3**	**1**	**3**	**0**	**16**	**42**
**Guidelines for patients with MDROs**	**Definition/designation of MDRO patients**		1	1	0	0	0	0	0	2	14
	**Protective measures**		2	2	1	1	0	1	0	7	14
		**Sum**	**3**	**3**	**1**	**1**	**0**	**1**	**0**	**9**	**28**
**Hand hygiene**	**Hand sanitizer**		2	0	2	2	0	2	1	9	14
	**Washing lotion**		2	1	1	2	0	2	1	9	14
	**Skin protection products**		1	0	0	2	0	0	0	3	14
	**Disposable towels**		2	2	2	2	2	2	1	13	14
		**Sum**	**7**	**3**	**5**	**8**	**2**	**6**	**3**	**34**	**56**
**Personal hygiene**	**Working clothes**		2	1	1	2	2	1	1	10	14

	**Hand jewelry and nails**		2	1	0	1	0	1	0	5	14
	**Food consumption**		1	1	1	2	0	1	0	6	14
	**Food storage**		1	1	1	2	0	1	0	6	14
	**Personnel changing rooms**		1	1	1	1	1	1	0	6	14
	**Laundry**		2	2	1	1	0	1	1	8	14
		**Sum**	**9**	**7**	**5**	**9**	**3**	**6**	**2**	**41**	**84**
**Protection of employees**	**Vaccinations**		1	2	1	0	2	2	1	9	14
	**Measures for pregnant/ immunosuppressed employees**		1	1	1	1	1	1	1	7	14
		**Sum**	**2**	**3**	**2**	**1**	**3**	**3**	**2**	**16**	**28**
**Protective clothing**	**Composition**		2	2	2	1	1	1	0	9	14
	**Use**		2	1	1	2	1	1	0	8	14
	**Storage and provision**		2	1	1	1	1	2	0	8	14
		**Sum**	**6**	**4**	**4**	**4**	**3**	**4**	**0**	**25**	**42**
**Medication**	**Preparation**		1	1	1	2	1	1	2	9	14
	**Storage**		2	1	2	2	2	2	2	13	14
	**Dating of open vials**		1	0	2	2	0	0	2	7	14
		**Sum**	**4**	**2**	**5**	**6**	**3**	**3**	**6**	**29**	**42**
**Guidelines and restrictions for antimicrobial use**	**Guidelines for antimicrobial use and dosing**		2	2	0	2	1	2	1	10	14
	**Restrictions for critically important antimicrobials**		2	2	0	1	0	2	0	7	14
		**Sum**	**4**	**4**	**0**	**3**	**1**	**4**	**1**	**17**	**28**
**Total score**			**55**	**35**	**33**	**48**	**19**	**33**	**20**	**243**	**476**

Abbreviations: IPC, Infection prevention and control; MDROs, multidrug resistant organisms.
